# Self-Rated Physical Health Among Working-Aged Adults Along the Rural-Urban Continuum — United States, 2021

**DOI:** 10.15585/mmwr.mm7105a1

**Published:** 2022-02-04

**Authors:** Danielle C. Rhubart, Shannon M. Monnat

**Affiliations:** ^1^Department of Biobehavioral Health, The Pennsylvania State University, State College, Pennsylvania; ^2^Lerner Center for Public Health Promotion and Department of Sociology, Syracuse University, Syracuse, New York.

Poor self-rated physical health is strongly associated with morbidity and premature mortality (*1*,*2*). Studies that are now a decade old report worse self-rated health among rural than among urban residents (*3*,*4*). Whether the rural disadvantage persists in 2021 is uncertain and the contributing factors to contemporary rural-urban variations in self-rated health are not known. Rural America is diverse by population size and adjacency to metropolitan areas, and rural populations vary demographically and socioeconomically. This analysis used data from the National Well-being Survey (NWS), a national sample of approximately 4,000 U.S. working-aged adults conducted during February and March 2021 to examine differences in self-rated physical health among residents of large urban; medium/small urban; metro-adjacent rural; and remote rural counties. Residents of medium/small urban, metro-adjacent rural, and remote rural counties had significantly higher probabilities of reporting fair/poor self-rated physical health than their large urban county peers. There were no significant differences by sex or race/ethnicity in self-rated physical health. Individual-level socioeconomic resources (including higher educational attainment, higher household income, and higher probability of employment) contributed to the advantage among residents of large urban counties. Although there is no single solution to reducing rural-urban health disparities, these findings suggest that reducing socioeconomic disparities is essential.

NWS is a national, cross-sectional, web-based survey of U.S. adults aged 18–64 years (working-aged adults). The survey was created and administered by the Syracuse University Lerner Center for Public Health Promotion during February and March of 2021. Recruitment was conducted by Qualtrics Panels, which uses a database of several million U.S. adults to recruit survey participants through nonprobability sampling.* Data collection included an oversample of rural residents to enable robust analyses. Poststratification demographic weights were used to allow generalizability to the broader U.S. working-aged population. Weights account for differential response by age, race/ethnicity, sex, educational attainment, and rural-urban residence. The NWS completion rate (i.e., completed surveys among those who viewed the landing page and the informed consent section) was 40.4%.

In addition to a standard set of demographic and socioeconomic questions, respondents were asked to answer the following standard self-rated physical health question: “In general, would you say your physical health is excellent, very good, good, fair, or poor?” Responses were dichotomized into fair/poor versus good, very good, or excellent. Survey responses were linked to county-level rural-urban continuum codes (RUCCs) from the U.S. Department of Agriculture Economic Research Service using county Federal Information Processing Standards codes.^†^ RUCCs were recoded into four categories: large urban counties (RUCC 1), medium/small urban counties (RUCCs 2 and 3), metro-adjacent rural counties (RUCCs 4, 6, and 8), and remote rural counties (i.e., not adjacent to a metro area) (RUCCs 5, 7, and 9).^§^ The recoded RUCC categories were used as the primary independent variable. Individual-level covariates included sex, age, race/ethnicity, marital status, household income, education, health insurance coverage, and employment status.^¶^ Given that data collection occurred approximately 1 year into the COVID-19 pandemic, models also control for respondents’ perceived impact of COVID-19 on their lives.

Among 4,014 persons in the original sample, 167 participants had missing information on variables of interest and their data were not used, resulting in a final analytic sample of 3,847. Descriptive statistics for self-rated physical health and model covariates are reported by rural-urban status. Logistic regression analyses predicting self-reported fair/poor physical health with clustered standard errors for states were used to calculate predicted probabilities of fair/poor physical health as a function of the rural-urban continuum and individual-level characteristics. All analyses were weighted with the poststratification weight and conducted using SAS software (version 9.4; SAS Institute). NWS survey and recruitment design were approved by the Syracuse University Institutional Review Board.

In the weighted sample of U.S. working-aged adults, the prevalence of reporting fair/poor physical health was significantly higher in medium/small urban (31.1%), metro-adjacent rural (40.2%), and remote rural (34.0%) counties than in large urban counties (23.4%) (Table 1). Rural-urban variation in several characteristics that might drive the observed variation in self-rated physical health was observed. Compared demographically with residents of large urban counties, those residing in metro-adjacent rural and remote rural counties were more likely to be female, older, and non-Hispanic White. In terms of socioeconomic differences, residents of metro-adjacent and remote rural counties were significantly more likely than residents of large urban counties to be on disability, have a high school diploma or less, be uninsured, and have annual household incomes <$25,000.

**TABLE 1:** Characteristics of U.S. adults aged 18–64 years, by rural-urban status* — National Well-being Survey, United States, 2021

Characteristic	County classification (weighted unadjusted %)					p-value
	Large urban (n = 1,770)	Medium/Small urban (n = 985)	Metro-adjacent rural (n = 687)	Remote rural (n = 405)	Chi-square statistic	
**Self-rated physical health**						
Fair/Poor	23.4	31.1	40.2	34.0	57.3	<0.001
**Sex**						
Female	45.3	54.1	62.0	62.7	57.9	<0.001
**Age group, yrs**						
18–29	23.5	24.1	20.3	18.0	19.0	0.004
30–49	46.1	40.7	41.3	48.5		
50–64	30.5	35.3	38.3	33.5		
**Race/Ethnicity**						
White, non-Hispanic	53.5	63.7	87.0	85.1	202.9	<0.001
Black, non-Hispanic	14.5	13.0	3.9	4.5		
Hispanic	22.9	16.0	5.0	6.0		
Other race	9.1	7.3	4.2	4.4		
**Marital status**						
Not married	55.8	58.7	58.3	56.4	2.7	0.564
**Employment status**						
Employed	61.0	52.6	44.8	45.2	78.2	<0.001
Unemployed	16.0	17.6	17.1	17.6		
Disability	6.5	10.4	16.3	15.1		
Retired/Homemaker/Student	16.5	19.3	21.9	22.0		
**Educational attainment**						
Bachelor’s degree or more	39.0	25.3	17.3	19.8	129.8	<0.001
Some college	29.0	33.4	32.2	32.5		
High school diploma or less	32.0	41.3	50.5	47.8		
**Health insurance**						
Uninsured	15.5	21.4	24.4	19.5	28.1	<0.001
**Household income, USD**						
≥50,000	50.2	38.4	30.7	27.0	127.4	<0.001
25,000–49,999	22.6	25.3	27.5	27.2		
<25,000	22.6	32.3	39.9	42.2		
Not reported	3.6	4.1	2.0	3.5		

**Abbreviation**: USD = U.S. dollars.

* Large urban counties are those in metropolitan areas of ≥1 million persons; medium/small urban counties are those in metropolitan areas of <1 million persons; metro-adjacent rural counties are those that are not in, but adjacent to, a metropolitan area; rural remote counties are those that are not in or adjacent to metropolitan areas.

Predicted probabilities of self-rated fair/poor physical health in the fully adjusted model indicate that the differences between large urban, medium/small urban, and remote rural counties were no longer statistically significant; however, a significantly higher probability of reporting fair/poor health persisted among residents of metro-adjacent rural counties (Table 2). Stepwise regression models demonstrated that the remote rural disadvantage observed in the unadjusted model is associated with lower income, lower educational attainment, and higher rates of disability in remote rural counties compared with those in large urban counties.

**TABLE 2:** Characteristics of U.S. adults aged 18–64 years, by unadjusted and adjusted probabilities of reporting fair/poor physical health* — National Well-being Survey, United States, 2021

Characteristic	No.	Unadjusted		Adjusted	
		%	p-value	%	p-value
**Overall**	**3,847**	**29.5**	**<0.001**	**27.4**	**<0.001**
**Rural-urban status^†^**					
Large urban	1,770	23.4	Ref	21.7	Ref
Medium/Small urban	985	31.1	<0.001	28.9	0.083
Metro-adjacent rural	687	40.2	<0.001	37.5	0.018
Remote rural	405	34.0	<0.001	31.6	0.575
**Sex**					
Male	1,897	—	—	23.1	Ref
Female	1,950	—	—	32.0	0.205
**Age group, yrs**					
18–29	882	—	—	26.9	Ref
30–49	1,732	—	—	24.7	0.562
50–64	1,233	—	—	31.5	0.407
**Race/Ethnicity**					
White, non-Hispanic	2,339	—	—	28.0	Ref
Black, non-Hispanic Black	494	—	—	25.9	0.076
Hispanic	710	—	—	27.8	0.826
Other	304	—	—	24.0	0.871
**Marital status**					
Married	1,730	—	—	20.6	Ref
Not married	2,117	—	—	33.0	0.079
**Employment status**					
Employed	2,268	—	—	18.3	Ref
Unemployed	567	—	—	37.6	<0.001
On disability	344	—	—	66.8	<0.001
Retired/Homemaker/Student	668	—	—	29.4	0.002
**Educational attainment**					
Bachelor’s degree or more	1,459	—	—	14.9	Ref
Some college	1,263	—	—	35.1	<0.001
High school degree or less	1,125	—	—	35.0	<0.001
**Health insurance**					
Insured	3,182	—	—	26.7	Ref
Uninsured	665	—	—	30.5	0.994
**Income, USD**					
≥50,000	1,777	—	—	15.4	Ref
25,000–49,999	901	—	—	36.1	<0.001
<25,000	1,040	—	—	41.2	<0.001
Not reported	129	—	—	20.8	0.747
c-statistic^§^	—	0.57	—	0.74	—

**Abbreviations:** Ref = referent group; USD = U.S. dollars.

* Logistic regression models are weighted and control for respondents’ self-report of impact of the COVID-19 pandemic on their lives and adjusted for clustered SEs for states.

^†^ Large urban counties are those in metropolitan areas of ≥1 million persons; medium/small urban counties are those in metropolitan areas of <1 million persons; metro-adjacent rural counties are those that are not in, but adjacent to, a metropolitan area; rural remote counties are those that are not in or adjacent to metropolitan areas.

^§^ The c-statistic is a measure of goodness of fit for binary outcomes and ranges from 0.5 to 1.0.

Several other characteristics were also associated with likelihood of self-reporting fair/poor health. Adjusted probabilities were higher among the following comparison groups: those who were unemployed (37.6%) or on disability (66.8%) versus those who were employed (18.3%), those with a high school diploma or less (35.0%) and some college (35.1%) versus those with a bachelor’s degree or more (14.9%), and those with household income <$25,000 (41.2%) or $25,000–$49,999 (36.1%) versus those with household income ≥$50,000 (15.4%).

## Discussion

Several important findings emerge from these analyses. Large differences in self-reported physical health exist among working-aged adults in the United States along the rural-urban continuum. Residents of medium/small urban, metro-adjacent rural, and remote rural counties are significantly more likely to self-rate their physical health as fair/poor than are residents of large urban counties. Given that self-rated health has been determined to be strongly associated with chronic health conditions and premature mortality, the limited city and rural disadvantage portends broader consequences for population health disparities. Recent studies report a large and growing rural mortality penalty (i.e., the long running trend of higher mortality rates in rural areas compared with those in urban areas) (*5*). A recent report from the National Academies of Sciences, Engineering, and Medicine (*6*) found that recent working-aged mortality increases have been most pronounced outside of large metropolitan areas. Adjusted models indicated that socioeconomic factors (e.g., lower education, lower income, lower rates of health insurance coverage, and lower levels of employment) account for much of the remote rural disadvantage in self-reported health. These findings are consistent with fundamental cause theory, wherein socioeconomic status affects disease outcomes through multiple risk pathways over time (*7*) and align with previous work illustrating a rural disadvantage in self-rated health that is in part tied to rural-urban differences in sociodemographic characteristics (*3*,*4*). The persistent metro-adjacent rural disadvantage might speak to the fact that counties in this category are more likely to be located in the South where a myriad of macro and structural factors produce worse health outcomes (e.g., lower access to care and higher place-level poverty rates) (*8*).

The findings in this report are subject to at least three limitations. First, the data are cross-sectional, and causality should not be inferred. Second, the data were collected approximately 1 year into the COVID-19 pandemic. Reports of self-rated physical health might have been affected by pandemic-related impacts. The models control for respondents’ self-perceived impact of the pandemic on their lives, but the findings should be viewed in the context of this enduring public health disruption. Finally, the sample is based on an opt-in web panel. Pew Research Center recently compared survey response estimates on 406 survey items for mail versus Internet-based responses and found that estimates differed by ≥5 percentage points on only nine items, all having to do with Internet access. Their report concluded that coverage bias associated with web surveys is modest for most kinds of measures (*9*).

A large body of research demonstrates that multiple factors are responsible for the worse rural health profile in the United States, suggesting that multiple policy strategies will be needed to address these disparities (*5*,*6*). Policies focused on reducing socioeconomic disparities, such as increasing the availability of livable wage jobs, especially for persons without a college degree, likely would address poor health outcomes in rural areas.

**Summary**

**What is already known about this topic?**

Self-rated physical health is strongly associated with morbidity and premature mortality. Decade-old studies report worse self-rated health among rural residents, but no recent reports exist on current rural-urban differences.

**What is added by this report?**

During 2021, working-aged adults in small/medium urban counties and rural counties reported worse physical health compared with residents of large urban counties. These differences are largely explained by differences in socioeconomic status (including lower educational attainment, household income, and probability of employment).

**What are the implications for public health practice?**

Policies addressing intersecting socioeconomic factors, including those that increase access to livable wage jobs, especially for those without a college degree, likely would reduce rural-urban health disparities.

## Footnotes

* Qualtrics Panels owns a database that includes data from several million U.S. adults who have agreed to participate in surveys. Participants are recruited using website intercept recruitment, member referrals, targeted email lists, gaming sites, customer loyalty web portals, permission-based networks, and social media. Names, addresses, and dates of birth are typically validated via third party-verification. For NWS data collection, panel members received an invitation with a hyperlink to NWS. Respondents were compensated in several different ways (e.g., airline miles or gift cards).

† https://www.ers.usda.gov/data-products/rural-urban-continuum-codes.aspx

§ Large urban counties are those in metropolitan areas of ≥1 million persons; medium/small urban counties are those in metropolitan areas of <1 million persons; metro-adjacent rural counties are those that are not in but adjacent to a metropolitan area; rural remote counties are those that are not in or adjacent to metropolitan areas.

¶ Respondents could select all that apply for the employment status question. Responses were recoded into four mutually exclusive groups: all those who indicated any disability; those who indicated unemployment, but no disability; those who indicated employment but no disability or unemployment; and those who did not indicate unemployment, employment, or disability (i.e., retired, homemakers, or students).
